# A RANDOMIZED CONTROLLED TRIAL OF SELF-HELP GUIDED BY LAY PROVIDERS FOR GENERALIZED ANXIETY DISORDER IN OLDER ADULTS

**DOI:** 10.1093/geroni/igad104.2142

**Published:** 2023-12-21

**Authors:** Philippe Landreville, Sébastien Grenier, Patrick Gosselin, Pierre-Hugues Carmichael

**Affiliations:** Université Laval, Quebec City, Quebec, Canada; Université de Montréal, Montreal, Quebec, Canada; Université de Sherbrooke, Sherbrooke, Quebec, Canada; Centre d’Excellence sur le Vieillissement de Quebec, Quebec, Quebec, Canada

## Abstract

Low use of professional help for anxiety disorders in older adults may be due in part to limited access to mental health services. Self-help guided by lay providers (LPs) could help improve access to treatment. This study evaluated the efficacy and long-term outcomes of self-help guided by LPs for generalized anxiety disorder (GAD) in older adults. Participants (≥ 60 years) with threshold or subthreshold GAD were randomly assigned to an intervention group (n = 75) or a wait-list control group (n = 75). Intervention group participants used a manual presenting readings and exercises based on principles of cognitive-behavioral therapy and received weekly support calls by LPs. Groups were similar in terms of sociodemographic characteristics and initially did not differ significantly on outcomes. At post-treatment, the intervention group had decreased significantly on various measures while the wait-list group remained stable: Generalized Anxiety Disorder 7-item (GAD-7): -4.8 vs -.03; Penn State Worry Questionnaire (PSWQ): -11.7 vs .07; Geriatric Anxiety Inventory (GAI): -5.3 vs -.07. Probability of having a diagnosis of threshold GAD decreased significantly more in the intervention group (79% to 16% vs 74% to 53%). Intervention group scores on the GAD-7, the PSWQ, and the GAI as well as probability of having a diagnosis of threshold GAD remained stable at 6- and 12-month follow-ups. Results provide evidence of the efficacy of self-help guided by LPs for GAD in older adults as well as of the maintenance of improvement following this treatment.

